# Draft Genome Sequence of *Leuconostoc mesenteroides* 213M0, Isolated from Traditional Fermented Mare Milk Airag in Bulgan Aimag, Mongolia

**DOI:** 10.1128/genomeA.00178-16

**Published:** 2016-03-31

**Authors:** Hidetoshi Morita, Hidehiro Toh, Kenshiro Oshima, Akiyo Nakano, Chihiro Hano, Saki Yoshida, Tsognemekh Bolormaa, Sedkhuu Burenjargal, Co Thi Kim Nguyen, Kosuke Tashiro, Kensuke Arakawa, Taku Miyamoto

**Affiliations:** a Graduate School of Environmental and Life Science, Okayama University, Okayama, Japan; b Medical Institute of Bioregulation, Kyushu University, Fukuoka, Japan; c Graduate School of Frontier Sciences, The University of Tokyo, Kashiwa, Chiba, Japan; d Department of Microbiology and Infectious Diseases, Nara Medical University, Kashihara, Nara, Japan; e School of Veterinary Science and Biotechnology, Mongolian State University of Agriculture, Ulaanbaatar, Mongolia; f Graduate School of Systems Life Sciences, Kyushu University, Fukuoka, Japan; g Faculty of Food Culture, Kurashiki Sakuyo University, Kurashiki, Japan

## Abstract

*Leuconostoc mesenteroides* 213M0 was isolated from traditional fermented mare milk airag in Bulgan Aimag, Mongolia. This strain produces a listericidal bacteriocin-like inhibitory substance. Here, we report the draft genome sequence of this organism.

## GENOME ANNOUNCEMENT

Airag is the most popular traditional fermented milk in Mongolia and Inner Mongolia of China. It is made by fermentation of mainly mare’s milk with various species of lactic acid bacteria. *Leuconostoc mesenteroides* is a species of lactic acid bacteria frequently detected in airag (1). The genus *Leuconostoc* represents a group within the low G+C content Gram-positive bacteria. We previously isolated *L. mesenteroides* 213M0, which produces a bacteriocin-like inhibitory substance presumably belonging to class IIa bacteriocins, from airag in Bulgan Aimag, Mongolia (2).

The 213M0 genome was paired-end sequenced using Illumina’s MiSeq platform. Genomic libraries containing 600 to 1,000 bp inserts were constructed and sequenced, yielding 3,153,464 sequences that provided 356-fold coverage from both ends of the genomic clones. The sequence reads were assembled using Newbler version 2.8 (Roche), and the assembled genome consists of 58 contigs with a total length of 2,032,849 bp. The draft genome of 213M0 contained 1,995 predicted protein-coding genes. Then, we compared the draft genome of 213M0 with that of *L. mesenteroides* 406 (accession no. BCMP01000000), which was isolated from another airag in Tuv Aimag, Mongolia (3, 4). Of the 1,995 protein-coding genes, 1,754 (88%) were shared by the both strains. The genome information of this species will be useful for further studies on food science and of its physiology, taxonomy, and ecology.

### Nucleotide sequence accession numbers.

The draft genome sequence for *L. mesenteroides* 213M0 has been deposited in the DDBJ/GenBank/EMBL database under the accession numbers BCMO01000001 to BCMO01000058.
